# Reduction of epileptiform activity in ketogenic mice: The role of monocarboxylate transporters

**DOI:** 10.1038/s41598-017-05054-0

**Published:** 2017-07-07

**Authors:** Linda S. Forero-Quintero, Joachim W. Deitmer, Holger M. Becker

**Affiliations:** Division of General Zoology, Department of Biology, University of Kaiserslautern, P.O. Box 3049, D-67653 Kaiserslautern, Germany

## Abstract

Epilepsy is a chronic neurological disorder that affects approximately 50 million people worldwide. Ketogenic diet (KD) can be a very effective treatment for intractable epilepsy. Potential mechanisms of action for KD have been proposed, including the re-balance among excitatory and inhibitory neurotransmission and decrease in the glycolytic rate in brain cells. KD has been shown to have an effect on the expression pattern of monocarboxylate transporters (MCT), however, it is unknown whether MCT transport activity is affected by KD and linked to the reduction of seizures during KD. Therefore, we studied the influence of KD on MCT transport activity and the role of MCTs during epileptiform activity. Our results showed a decrease in the epileptiform activity in cortical slices from mice fed on KD and in the presence of beta-hydroxybutyrate. KD increased transport capacity for ketone bodies and lactate in cortical astrocytes by raising the MCT1 expression level. Inhibition of MCT1 and MCT2 in control conditions decreases epileptiform activity, while in KD it induced an increase in epileptiform activity. Our results suggest that MCTs not only play an important role in the transport of ketone bodies, but also in the modulation of brain energy metabolism under normal and ketogenic conditions.

## Introduction

Epilepsy is characterized by the occurrence of unprovoked seizures, which result from excessive, synchronous, abnormal electrical firing patterns of a population of neurons^1, 2^. Normal brain function is dependent upon the balance between inhibition and excitation in neuronal circuits. Disruption of this balance, by increasing excitatory processes^3–5^ or by decreasing or losing inhibitory mechanisms^6–8^, leads to uncontrolled neuronal activity or epileptiform activity, producing augmented glutamate release, which in turn is sensed by astrocytes via metabotropic glutamate receptors (mGluR), resulting in changes in intracellular Ca^2+^ levels^9, 10^.

Epilepsy affects approximately 1% of the population worldwide, of which more than ¼ of patients remain pharmacoresistant. Ketogenic diet (KD) is a treatment employed in patients with refractory epilepsy, particularly children. KD is a high-fat diet with adequate protein and low carbohydrates. It was developed to mimic the physiological effects of fasting without starvation, a treatment reported to control seizure activity^11–13^. While it is known that KD reduces the incidence of seizures, the mechanisms of action are not completely understood^14, 15^. KD forces the body to metabolize fat rather than carbohydrates. The oxidation of fatty acids produces acetyl-CoA, which cannot be entirely used in the Krebs cycle, and is shunted into the ketogenesis pathway to produce the ketone bodies (KB) acetoacetate (ACA) and β-hydroxybutyrate (BHB), which are released into the blood stream, from where they can enter the brain tissue and brain cells via monocarboxylate transporter 1 (MCT1). MCTs transport high energy metabolites like lactate and pyruvate, but also ACA and BHB together with H^+^ in a stoichiometry of 1:1. In neurons transport is mediated through MCT1 and MCT2^16–18^, in astrocytes through MCT1 and MCT4^19, 20^. Recent studies reported a decrease or even loss of MCT1 and MCT2 expression in the micro vessels of patients and animal models with temporal lobe epilepsy (TLE)^21–23^. These changes in the cellular distribution of MCTs suggest that clearance of brain lactate, as well as the uptake and release of KB, may be impaired in TLE. Contrary, it has been shown that KD can lead to an increase in MCT1 expression level in rat brain^24, 25^.

In the present study, we investigated the influence of KD on MCT transport activity using H^+^-imaging, and its influence on epileptiform activity using Ca^2+^-imaging. The experiments were performed in cortical astrocytes from mice fed either control or ketogenic diets (CD and KD). We observed that KD led to a robust increase in KB and lactate flux, mediated by increased MCT1 expression levels. Epileptiform activity was dramatically decreased in slices from mice following KD, but this effect was reversed by inhibition of MCT1 and MCT2. In contrast, inhibition of MCT1 and MCT2 in slices from mice following CD led to a decrease in epileptiform activity. Our results suggest that MCT1 and MCT2 not only play an important role in the transport of KB under ketogenic conditions, but also in the modulation of lactate movement between astrocytes and neurons under normal and ketogenic conditions.

## Results

### Ketogenic diet decreases induced neuronal epileptiform activity in cortical slices

Our main objective was to study the influence of KD on MCT transport activity during epileptiform activity. We considered two groups of mice and fed one group with a CD and the other one with a KD. The animals body weight and food intake were measured to ensure that both groups had the same conditions before brain cortex extraction.

For the first three weeks mice on KD had a significantly lower gain in body weight as mice on CD. However, after that time period both groups gained weight equally well and no significant differences in body weight could be observed during the time period where experiments were carried out (Fig. S1a). Food intake (F.I.) was also significantly less in mice on KD as compared to the group on CD during the first 1.5 weeks. However, the caloric consumption in both groups was comparable from that point on (Fig. S1b). Due to the initial differences in body weight and food intake, preparation of acute cortical slices was performed in mice at the age between 60 and 85 days (4 weeks in treatment).

**Figure 1:**
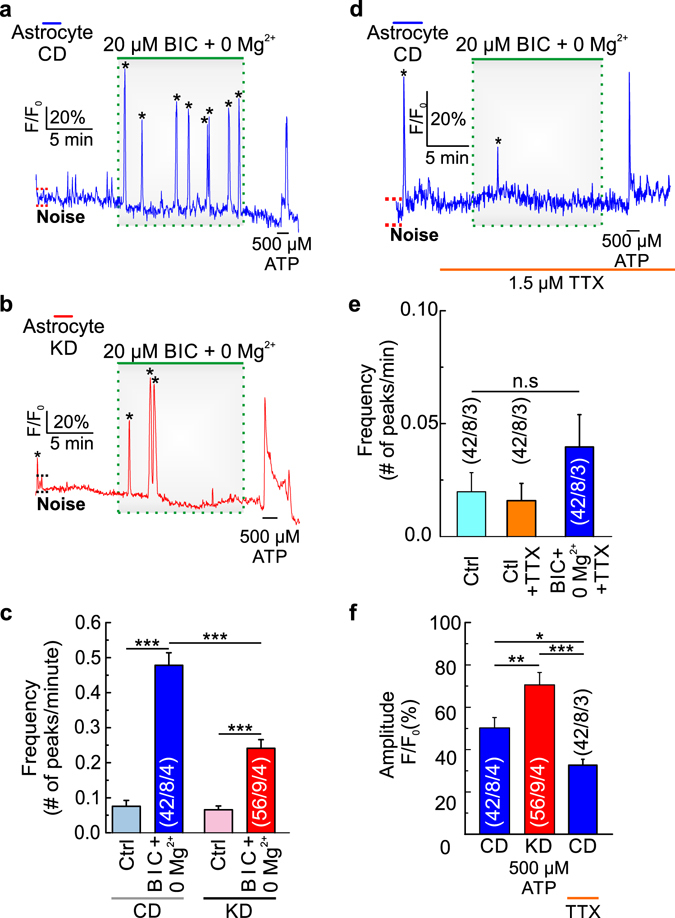
Epileptiform activity is reduced by the action of the ketogenic diet in cortical slices. Changes in astrocytic Ca^2+^ signals, as induced by neuronal epileptiform activity (0 Mg^2+^/BIC) in cortical slices in CO_2_/HCO_3_^−^-buffer from mice that followed a control (**a**) and a ketogenic diet (**b**) in the absence of TTX, and from mice fed with a control diet in the presence of TTX (**d**). One short pulse of ATP was given at the end of each measurement to verify astrocytic Ca^2+^ response. Frequency of the peaks occurred spontaneously (ctrl; light blue and light red bars), and induced by BIC/0Mg^2+^ in CO_2_/HCO_3_^−^-buffer, in slices from mice that followed control (blue bar) and ketogenic (red bar) diets in the absence of TTX (**c**), and in slices from control mice in the presence of TTX (**e**). Amplitude of the peaks induced by ATP in astrocytes from control (blue bar) and ketogenic mice (red bar) in the absence of TTX, and from control mice in the presence of TTX (**f**). Statistical values are presented as means ± S.E.M. Significance was tested with paired Student’s t-test. Number of astrocytes/slices/mice used are indicated in the bar plots.

To investigate the impact of epileptiform activity on slices from both dietary groups, we monitored intracellular Ca^2+^ changes in astrocytes in acute cortical slices from mice that followed CD and KD, by exposing the samples to Mg^2+^-free standard buffer, supplemented with 20 µM bicuculline (0 Mg^2+^/BIC) for 12 min (Fig. 1a,b). We used the frequency of Ca^2+^ transients in astrocytes as measure for neuronal activity. The frequency of the Ca^2+^ transients induced by 0 Mg^2+^/BIC in slices from mice following KD was significantly reduced compared to slices from animals fed with CD (Fig. 1c). This confirms that KD led to a reduction in epileptiform activity in cortical slices of adult mice. Furthermore, the frequency of Ca^2+^ transients evoked spontaneously were significantly smaller than the frequency of Ca^2+^ transients elicited by 0 Mg^2+^/BIC in slices from mice following CD and KD, which indicates that the Ca^2+^ transients evoked during 0 Mg^2+^/BIC application were indeed a result of induced epileptiform activity. There was no significant difference in the frequency of spontaneous Ca^2+^ transients before application of 0 Mg^2+^/BIC between slices from mice following CD and KD.

To confirm that 0 Mg^2+^/BIC induced epileptiform activity by direct and exclusive action in neurons, 0 Mg^2+^/BIC was applied in the presence of TTX, which prevents action potential generation (Fig. 1d). Indeed application of 0 Mg^2+^/BIC in the presence of TTX did not result in a significant increase in astrocytic Ca^2+^ transients (Fig. 1e), which confirmed that the protocol used in the present study induced epileptiform activity by direct and exclusive action on neurons, which was further confirmed using the same protocol in pure astrocyte cultures, which showed no change in the frequency of Ca^2+^ transients (Fig. S2). At the end of each measurement, a short pulse of ATP was applied to check cell viability and to confirm that the astrocytes are still able to elicit Ca^2+^ responses (Fig. 1a,b,d,f).

**Figure 2:**
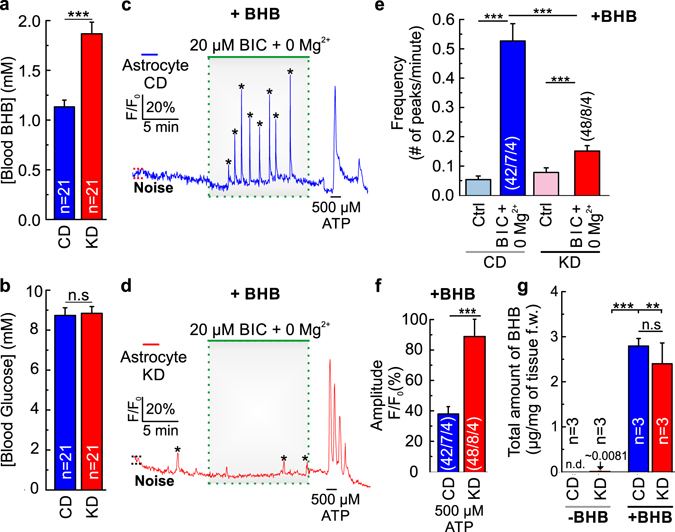
The presence of BHB does not affect epileptiform activity in slices from control mice, but decreases further the induced activity in slices of ketogenic mice. Blood levels of (**a**) BHB and (**b**) glucose in control (blue bars) and ketogenic (red bars) mice. Changes in astrocytic Ca^2+^ signals, as induced by epileptiform activity (0 Mg^2+^/BIC) in cortical slices in CO_2_/HCO_3_^−^-buffer in the presence of BHB, from mice that followed a control (**c**) and a ketogenic diet (**d**). Frequency of the peaks occurred spontaneously (ctrl; light blue and light red bars) and induced by 0 Mg^2+^/BIC in CO_2_/HCO_3_^−^-buffer in the presence of BHB, in slices from mice that followed control (blue bar) and ketogenic (red bar) diets (**e**). Amplitude of the peaks induced by ATP in astrocytes from control (blue bar) and ketogenic mice (red bar) in the presence of BHB (**f**). Mass spectrometry-based quantification of the total amount of BHB in slices from control (blue bars) and ketogenic (red bars) mice, in the absence and in the presence of BHB (**g**). Statistical values are presented as means ± S.E.M. Significance was tested with paired Student’s t-test. Number of astrocytes/slices/mice used are indicated in the bar plots.

The concentration of BHB in the blood was significantly increased in mice that were fed with KD as compared to animals that followed CD, with a mean concentration of 1.87 ± 0.32 mM and 1.13 ± 0.06 mM for KD and CD mice, respectively (Fig. 2a), while blood glucose concentration did not differ significantly between both groups (Fig. 2b). To study whether BHB has a direct effect on induced epileptiform activity, we performed the same experiments as described in Fig. 1 in the presence of 2 mM BHB (Fig. 2c,d). The frequency of the Ca^2+^ transients elicited by application of 0 Mg^2+^/BIC in the presence of BHB in slices from mice fed on CD increased significantly more than in slices from mice fed on KD (Fig. 2e), similar as in the same experiments performed in the absence of BHB (Fig. 1c). No reduction in the frequency of the Ca^2+^ transients was observed in slices from mice following CD in the presence of BHB as compared to slices from mice fed with CD in the absence of BHB (Figs 1c and 2e). However, the frequency of the Ca^2+^ transients in slices from mice in KD was slightly but significantly reduced in the presence of BHB (0.15 ± 0.017 peaks/min) as compared to KD in the absence of BHB (0.24 ± 0.024 peaks/min) (Figs 1c and 2e). Cell viability was checked by the response to a pulse of ATP at the end of each measurement (Fig. 2c,d). The amplitude of the evoked peaks was significantly higher in slices from mice following KD as compared to slices of animals fed on CD in the presence of BHB (Fig. 2f), presumably due to the lower frequency of Ca^2+^ transients.

Mass spectrometric (MS) analysis showed that tissue concentrations of BHB were beyond detection limit in mice following CD, while tissue samples from mice following KD contained a very small concentration of 0.0081 ± 0.0024 µg BHB/mg of tissue, when the samples were prepared in the absence of BHB (Figs 2g, S3a). For the samples prepared in the presence of 2 mM BHB, the total amount of BHB was 2.80 ± 0.17 µg BHB/mg of tissue for slices from mice following CD and 2.40 ± 0.47 µg BHB/mg of tissue for mice following KD (Figs 2g, S3b). These concentrations were relatively close to the amount of BHB added to the medium during slices preparation (2 mM or 3.47 µg BHB/mg of tissue). These measurements indicate that BHB was almost entirely washed out of the slices from mice following KD after three hours of incubation in BHB-free saline. Determination of lactate concentrations by MS showed no significant difference between slices from mice fed on CD or on KD, and incubation in BHB-containing or BHB-free saline, respectively, with an average concentration of ~0.24 µg lactate/mg of tissue (Fig. S3c–e).

Taken together the results indicate that KD dramatically decreases chemically-induced epileptiform activity in acute cortical slices. Additionally, acute removal of BHB from the medium does decrease, but not abolish, the dampening effect, induced by several weeks of KD, while short term exposure to BHB in slices from mice that followed CD did not reduce epileptiform activity. The results are consistent with the neuroprotective effect attributed to KD^13–15, 26–28^, and recurrence of seizures when the treatment is stopped^13, 29^. However, the observation that acute withdrawal of BHB reduces the dampening effect under KD, but acute application of BHB does not induce neuroprotection under CD, indicates that neuroprotection by KD is based on at least two mechanisms, one depending on the acute presence of BHB and one long-term effect that could not be induced by acute application of BHB.

### Transport capacity for KB and lactate is increased in cortical astrocytes during ketogenic diet

**Figure 3:**
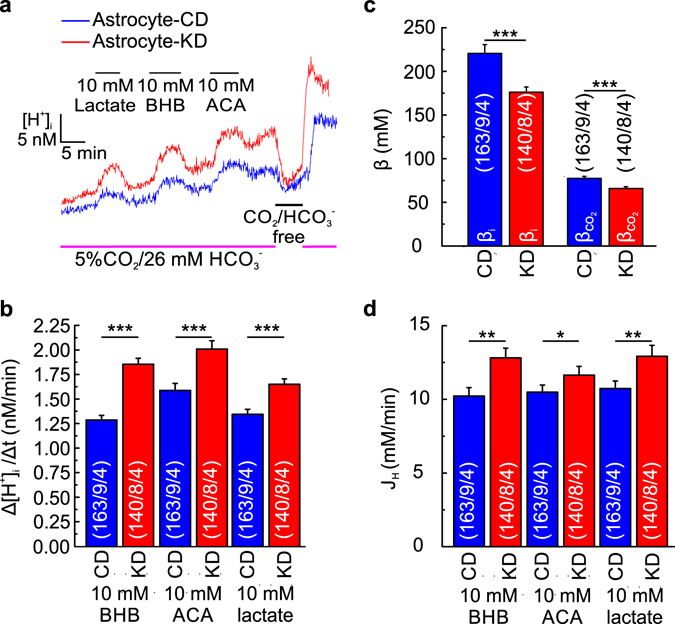
Ketogenic diet increases MCT transport activity in cortical astrocytes. (**a**) Original recordings of the change in [H^+^]_i_, as induced by application of 10 mM lactate, BHB and ACA in cortical astrocytes from mice fed with control diet (CD, blue traces) and ketogenic diet (KD, red traces) in 5% CO_2_/26 mM HCO_3_^−^-buffer, then a pulse of CO_2_/HCO_3_^−^-free buffer was given. (**b**) ${\rm{\Delta }}{[{{\rm{H}}}^{+}]}_{{\rm{i}}}/{\rm{\Delta }}{\rm{t}}$, as induced by application of 10 mM of BHB, ACA and lactate in CO_2_/HCO_3_^−^-buffer in cortical astrocytes from CD (blue bars) and KD (red bars). (**c**) Intrinsic (β_i_) and CO_2_/HCO_3_^−^-dependent $({{\rm{\beta }}}_{{{\rm{CO}}}_{2}})$ buffer capacity in cortical astrocytes from CD (blue bars) and KD (red bars). (**d**) J_H_, as induced by 10 mM of BHB, ACA and lactate in CO_2_/HCO_3_^−^-buffered solution in astrocytes from CD (blue bars) and KD (red bars). Statistical values are presented as means ± S.E.M. Significance was tested with a paired Student’s t-test. Number of astrocytes/slices/mice are indicated in the bar plots.

To study the effect of KD on MCT transport activity, we measured the rate of change in intracellular H^+^ concentration $({\rm{\Delta }}{[{{\rm{H}}}^{+}]}_{{\rm{i}}}/{\rm{\Delta }}{\rm{t}})$, during application of 10 mM of BHB, ACA and lactate in astrocytes from acute cortical slices prepared from mice that followed CD and KD, respectively (Fig. 3a). Astrocytes from mice following KD were slightly more acidic than astrocytes from mice following CD (ΔpH = 0.027 ± 0.019; p ≤ 0.05). KB/lactate-induced acidification was significantly increased in astrocytes from mice following KD as compared to mice following CD, which indicates an increase in MCT transport activity in cortical astrocytes from mice fed with KD (Fig. 3b). Interestingly both intrinsic (β_i_) and CO_2_/HCO_3_^−^-dependent buffer capacity (${{\rm{\beta }}}_{{{\rm{CO}}}_{2}}$) were significantly reduced in mice following KD (Fig. 3c), however net H^+^ flux (J_H_), as induced by KB and lactate was still significantly increased in astrocytes from mice on KD.

**Figure 4:**
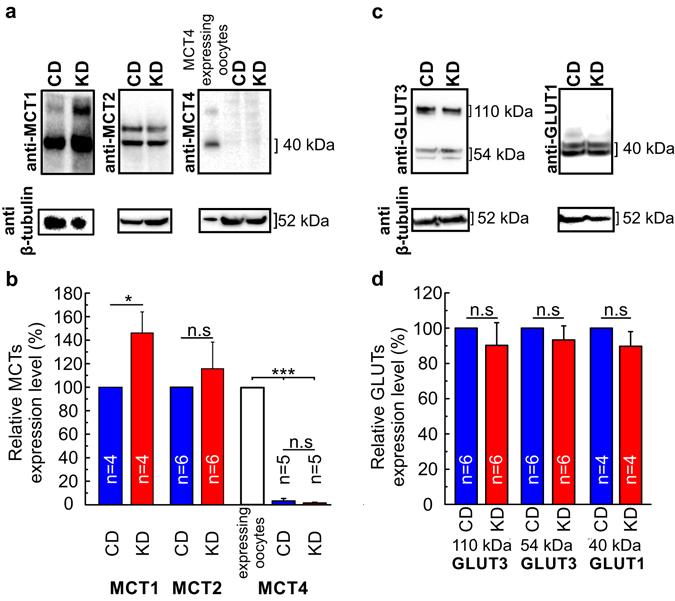
Ketogenic diet increases MCT1 expression level in mouse brain cortex. Western blot analysis in cortex from mice fed with control (CD) and ketogenic diet (KD) against MCT1, MCT2 and MCT4 (**a**), and GLUT3 and GLUT1 (**b**), β-tubulin is shown as loading control in each blot. The blots were cropped for better clarity. The full-length blots are shown in Fig. S4. Quantification of the relative expression levels for MCT (**c**) and GLUT (**d**) proteins. Results were normalized to the CD protein concentration on the same blot or to the concentration of MCT4 obtained for MCT4-expressing oocytes (only for MCT4). Statistical values are presented as means ± S.E.M and significance was tested with a paired Student’s t-test. Number of mice used are indicated in the bar plots.

To investigate whether the increase in MCT transport activity during KD is due to an increase in the transporters expression level, we quantified expression levels of MCT1, MCT2, and MCT4 in the brain cortex of mice fed on CD and KD using western blot (Fig. 4a). Protein bands were observed for MCT1 and MCT2, but not for MCT4 in samples from mice fed on CD and KD. MCT4 antibody efficacy was demonstrated with MCT4-expressing oocytes, where a band was observed at 40 kDa (Fig. 4a). The full-length blots are shown in Fig. S4a–c. Relative expression level of MCT1 increased in brain cortex of mice fed with KD to 146.12 ± 17.88%, as compared to the samples from mice following CD (Fig. 4b). MCT2 expression was not significantly altered by KD (115.69 ± 22.69%), as compared to the samples from mice following CD. Since a change in MCT expression levels indicates an alteration in cell metabolism, we also investigated the expression level of the two glucose transporters GLUT1 and GLUT3. Neuron-specific GLUT3 was expected to be detected at 54 kDa, but two bands were observed, one at 54 kDa and the other one around 110 kDa (Fig. 4c). These might be the monomeric and dimeric forms of the transporter, respectively. Thus, both bands were considered for quantification. Astrocyte-specific GLUT1 was detected at 40 kDa (Fig. 4c). No significant difference in the expression levels were found in mouse brain cortex for GLUT3 and GLUT1 between samples from mice that were fed on CD and KD (Fig. 4d). The full-length blots are shown in Fig. S4d,e.

**Figure 5:**
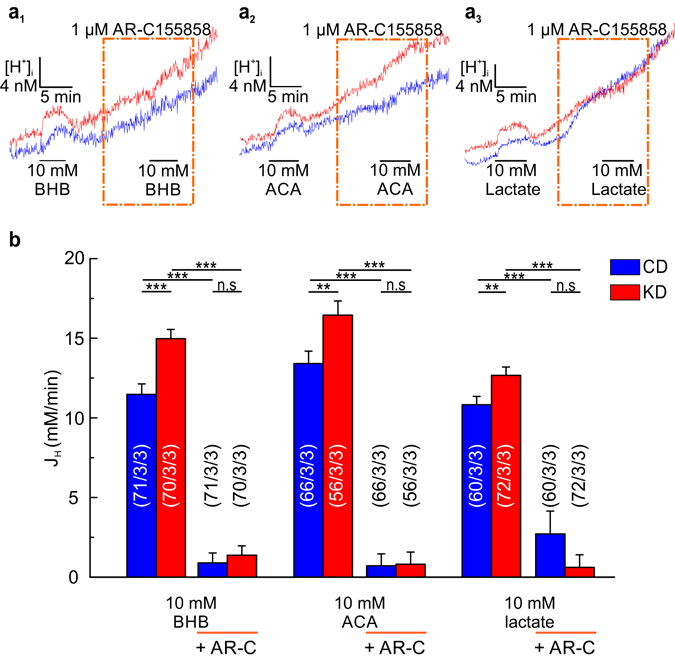
Enhanced transport activity under ketogenic conditions is mediated by MCT1. Original recordings of the change in [H^+^]_i_, as induced by application of (**a**_**1**_) 10 mM BHB, (**a**_**2**_) 10 mM ACA, and (**a**_**3**_) 10 mM lactate in cortical astrocytes from mice fed with control diet (CD, blue traces) and ketogenic diet (KD, red traces) in 5% CO_2_/26 mM HCO_3_^−^-buffer in the absence and in the presence of 1 µM of AR-C155858 (dashed orange boxes). (**b**) J_H_, as induced by 10 mM of BHB, ACA and lactate in CO_2_/HCO_3_^−^-buffered solution in astrocytes from CD (blue bars) and KD (red bars) in the absence and in the presence of AR-C155858. Statistical values are presented as means ± S.E.M and significance was tested with a paired Student’s t-test. Number of astrocytes/slices/mice are indicated in the bar plots.

In order to elucidate, whether transport of KB and lactate was also mediated by MCT4 in astrocytes, we measured changes in the intracellular H^+^ concentration in cortical astrocytes from mice following CD and KD, respectively, during application of BHB, ACA, and lactate in the absence and in the presence of 1 µM of AR-C155858 (Fig. 5a_1_, a_2_ and a_3_), a potent inhibitor of MCT1 and MCT2, but not of MCT4^30^. Calculation of the net H^+^ flux (J_H_) showed that transport of KB and lactate were almost completely blocked by AR-C155858 in astrocytes from mice fed either on CD or KD. This indicates that MCT4 is not involved in the transport of KB and lactate in cortical astrocytes from mice under both conditions, and that the increase in transport activity, observed in mice following KD, is most likely mediated by an increase in the expression level of MCT1.

### Inhibition of MCT1 and MCT2 decreases epileptiform activity under control conditions, but increases epileptiform activity under ketogenic conditions

**Figure 6:**
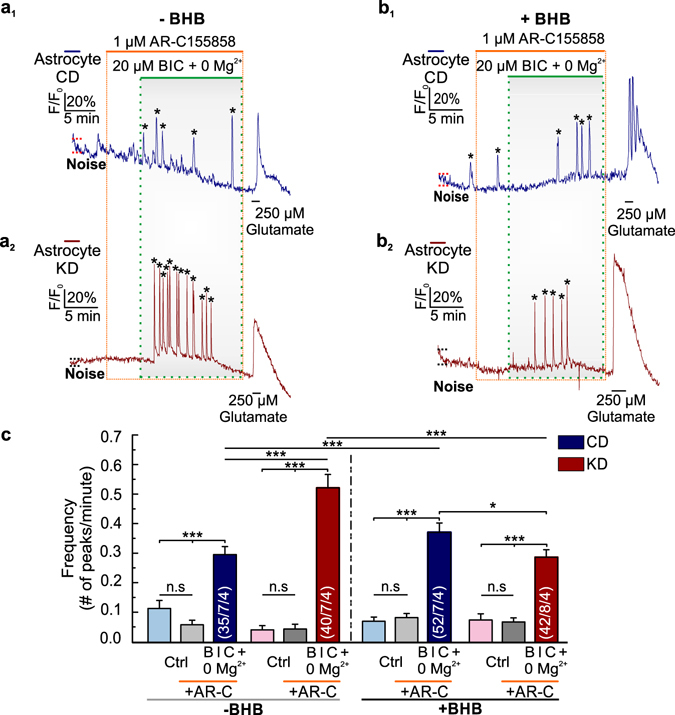
Inhibition of MCT1 and MCT2 in cortical slices decreases epileptiform activity in control mice, while this inhibition increases epileptiform activity in ketogenic mice. Changes in astrocytic Ca^2+^ signals, as induced by epileptiform activity (dotted green boxes) in cortical slices in the presence of 1 µM of AR-C155858 (dashed orange boxes) from control (dark blue traces) and ketogenic (wine red traces) mice in CO_2_/HCO_3_^−^-buffered solution, in the absence (**a**_**1**_ and **a**_**2**_) and the presence (**b**_**1**_ and **b**_**2**_) of BHB. One short pulse of 250 µM L-glutamate was given at the end to verify astrocytic calcium response. (**c**) Frequency of the peaks occurred spontaneously in the absence (light blue and light red bars) and the presence of AR-C155858 (light and dark grey bars), and induced by 0 Mg^2+^/BIC (dark blue and wine red bars) in CO_2_/HCO_3_^−^-buffer, in the absence and the presence of BHB.

To identify the role of MCTs during epileptiform activity under ketogenic and normal metabolic conditions we monitored intracellular Ca^2+^ transients in astrocytes of cortical slices from mice fed on CD and KD, respectively. Epileptiform activity was induced by exposing the slices to 0 Mg^2+^/BIC in the presence of 1 µM AR-C155858, without and with added BHB. At the end of each experiment, a pulse of glutamate was applied to check for cell viability and Ca^2+^ response capability (Fig. 6a_1_,a_2_,b_1_,b_2_). Inhibition of MCT1 and MCT2 by AR-C155858 in the absence of BHB in slices from mice following KD led to a significant increase in the frequency of the Ca^2+^ transients induced by 0 Mg^2+^/BIC (Fig. 6a_2_), as compared to slices from mice following CD in the absence and in the presence of BHB (Fig. 6a_1_,b_1_), and as compared to slices from mice following KD in the presence of BHB (Fig. 6b_2_,c). In contrast, in the presence of BHB, the frequency of Ca^2+^ transients during inhibition of MCT1 and MCT2 in slices from mice following KD was still significantly smaller as compared to slices from mice following CD (Fig. 6c).

The results show that removal of BHB from the medium together with inhibition of MCT1 and MCT2 in cortical slices led to a significant increase in the epileptiform activity in slices from mice following KD. These results suggest that MCT1 and MCT2 are essential in the uptake of KB during KD to decrease chemically-induced epileptiform activity, and continuous exposure to BHB is necessary to maintain the efficacy of KD.

**Figure 7:**
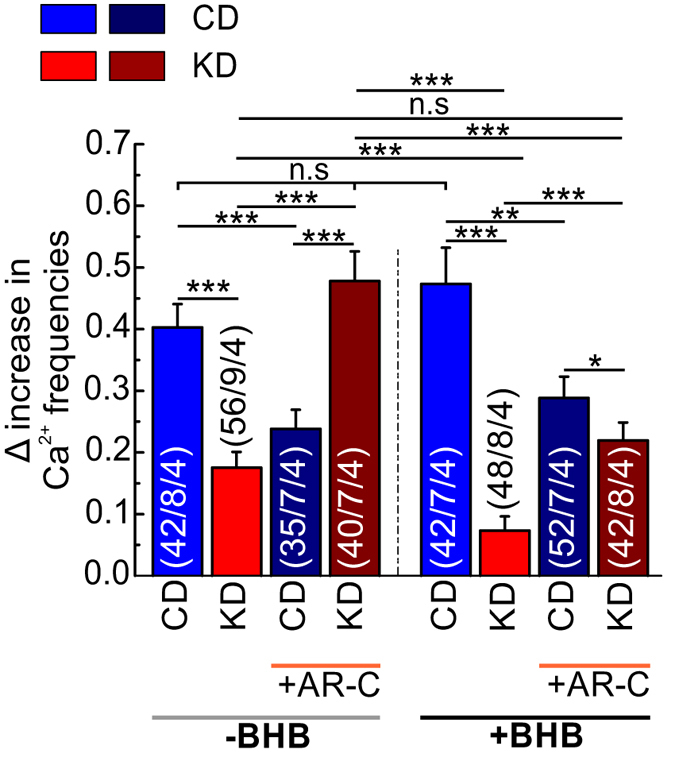
Net changes in the frequency of Ca^2+^ transients elicited by 0 Mg^2+^/BIC in cortical slices from mice following control and ketogenic diets. ΔCa^2+^ of the frequency of the peaks occurred spontaneously and induced by 0 Mg^2+^/BIC in the absence (blue and red bars) and the presence (dark blue and wine red bars) of 1 µM AR-C155858, in control and ketogenic cortical slices, respectively. Statistical values are presented as means ± S.E.M. Significance was tested with a paired Student’s t-test. Number of astrocytes/slices/mice used are indicated in the bar plots.

To compare the net effects of CD and KD, in the presence and absence of BHB, and following inhibition of MCT transport activity by AR-C155858, we calculated the difference (Δ) in the increase of Ca^2+^ frequency, induced by application of 0 Mg^2+^/BIC, for each condition, by subtracting the frequency of the spontaneous Ca^2+^ transients from the frequency of the Ca^2+^ transients elicited by application of 0 Mg^2+^/BIC (Fig. 7). (1) As compared to mice on CD, epileptiform activity decreased in mice on KD in the absence of BHB and AR-C155858, in the presence of BHB and absence of AR-C155858, and in the presence of BHB and AR-C155858. However, in the absence of BHB and presence of AR-C155858 epileptiform activity increased. (2) Inhibition of MCT transport activity by AR-C155858 in mice following KD resulted in an increase in epileptiform activity, both in the presence and absence of BHB, while in mice on CD inhibition of MCT activity resulted in a decrease. (3) Addition of BHB to the medium resulted in a significant decrease in epileptiform activity in mice fed on KD, both in the presence and absence of AR-C155858, while in mice fed on CD, addition of BHB had only minor effects.

These results suggest, on the one hand, that the uptake of KB via MCT1/MCT2 during KD together with the constant exposure to BHB contribute effectively to the reduction of neuronal activity as induced by 0 Mg^2+^/BIC. On the other hand, inhibition of MCT1 and MCT2 in slices from mice on CD during application of 0 Mg^2+^/BIC led to a reduction of epileptiform activity, which indicates that disruption of the lactate shuttle between astrocytes and neurons could lead to a reduction in epileptiform activity under control conditions.

## Discussion

KD has been largely used as an effective treatment for intractable epilepsy. However, the molecular mechanisms under which KD diminishes the incidence of seizures are still poorly understood. Nevertheless, some mechanisms of action have been suggested^14, 15^, including a potential role of MCTs in the reduction of epileptiform activity during KD, as shown in the present study. Here, we unravel the influence of KD on MCT transport activity, and the role of MCT in the decrease of epileptiform activity. Neuronal activity was assessed by measuring the frequency of astrocytic Ca^2+^ transients as previously shown^31–34^. We report that a KD dampens neuronal activity in brain cortex of adult mice during induced epileptiform activity. The frequency of Ca^2+^ transients during epileptiform activity is significantly reduced in cortical astrocytes from animals fed on KD. Contrary, removal of BHB from the medium several hours before the experiment partially reduced the neuroprotective effect induced with KD, but did not affect the frequency of Ca^2+^ transients in cortical astrocytes from mice on CD. This correlates with a reversion of the neuroprotective effect that has been observed in clinical models when KD is discontinued^13, 29^. BHB is one of the KB produced after fatty acids oxidation during KD, and it has been shown that BHB can directly interact with the Cl^−^ binding site of VGLUT and thereby reduce synaptic glutamate release. Our mass spectrometry measurements showed that slices prepared in the absence of BHB contain almost no residual BHB. This suggests that the increase in epileptiform activity observed in the absence of BHB might be attributable to a reactivation of VGLUT after removal of BHB from the medium. Nevertheless, the fact that the frequency of Ca^2+^ transients does not reach values similar to the control in the absence of BHB indicates that despite the reduction in BHB total concentration, additional effects induced by KD still remain after BHB removal.

Interestingly, inhibition of MCT transport activity by AR-C155858 in mice on KD resulted in an increase in epileptiform activity, while in mice on CD, inhibition of MCT transport activity led to a decrease of neuronal activity. These results suggest that MCT1 and/or MCT2 are important in the uptake of KB, and indirectly in the reduction of epileptiform activity during KD, while the disruption of the astrocyte to neuron lactate shuttle (ANLS) by inhibition of MCT1/MCT2 reduces epileptiform activity in control conditions. KD also increases proton-coupled transport of lactate and KB by an increase in the MCT1 expression level. MCT1 is the major isoform in cortical astrocytes in control and ketogenic conditions, as shown by the inhibition of MCT transport activity with AR-C155858. In line with this result, it was shown in earlier studies that KD induces up-regulation of MCT1 in rats^24, 25^. Although MCT4 is reported to be expressed in the brain cortex of rats^18, 35^, the results presented here suggest very low or no expression of MCT4 in the brain cortex of adult mice on CD or KD, since neither MCT4 protein nor physiological activity of MCT4 were detected. This variation in MCT4 expression may be attributable to the difference in species and age of the animals^36^.

**Figure 8:**
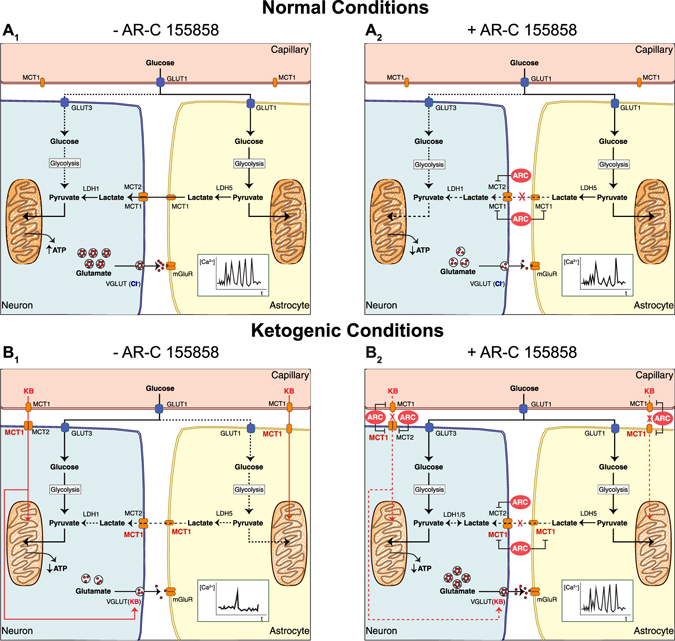
Schematic model of the mechanism of action for MCTs during epileptiform activity in CD and KD. During normal conditions, astrocytes take up glucose to produce lactate, and then export it to the neurons via MCT. During epileptiform activity, neurons increase their energetic demand, which leads to an increase in the ATP production and glutamate release (**A**_**1**_). When MCT1 and MCT2 transport activity are inhibited by AR-C155858 (ARC), the ANLS is disrupted, leading to a decrease in the ATP production, and consequently to a decrease in epileptiform activity (**A**_**2**_). Under ketogenic conditions, KB concentration increases, leading to a reduction in the glycolytic rate in the astrocytes, which in turn reduces lactate and ATP production, in astrocytes and neurons, respectively. Neurons also take up KB, and now the availability of glucose increases for them. KB enter to the mitochondria and activate the TCA cycle, in addition, they modulate the glutamate release from VGLUTs, which leads to a dampening in the epileptiform activity (**B**_**1**_). Inhibition of MCT1 and MCT2 with AR-C155858 inhibits the uptake of KB and lactate via MCT in astrocytes and neurons. Epileptiform activity increases because KB cannot longer modulate VGLUTs, and due the conversion of the remaining lactate into pyruvate in the neurons (**B**_**2**_). See more details in the text.

Based in the results obtained in the present study and considering that (1) glutamate release via VGLUT in neurons is reduced by action of KB^37^, (2) astrocytic glucose consumption is strongly inhibited by BHB^38^, (3) inhibition of lactate dehydrogenase (LDH) suppresses seizures^39^, we propose a hypothetical model of the role of MCTs during epileptiform activity in CD and KD (Fig. 8). Under normal conditions, glucose supplies energy to neurons and astrocytes, which is transported through GLUT3 and GLUT1, respectively. In the astrocytes, glucose is converted to lactate via glycolysis, which is then extruded via MCT1 and taken up by the neurons through MCT1 and MCT2. Both fuels meet the increased neuronal energy demand, induced by epileptiform activity under normal conditions. Thus, ATP production is maintained and consequently hyperexcitability is prolonged, leading to an increased vesicular glutamate release, which is fuelled by VGLUTs. Glutamate in turn is sensed by mGluR in the astrocytes, triggering an increase in its cytosolic [Ca^2+^] (Fig. 8A_1_). Under normal conditions, inhibition of MCT1 and MCT2 leads to a decrease in the epileptiform activity, mediated by a disruption of the ANLS, which may lead to a reduction in the ATP production and consequently reduced glutamate release (Fig. 8A_2_). This idea agrees with a recent study^39^, in which the authors linked the inhibition of LDH to the suppression of seizures. Inhibition of LDH reduces ATP production, which activates K_ATP_ channels and in turn leads to a decrease in neuronal excitability^40^.

During ketogenic conditions, glucose and KB are available in the blood stream. Under these conditions MCT1 is up-regulated, which improves KB uptake into astrocytes and likely also in neurons. KB are efficiently metabolized in the astrocytic mitochondria, leading to a reduction in glucose uptake^38^ and consequently decreased production and export of lactate. For neurons, this results in decreased availability of lactate but increased availability of glucose, which is imported and metabolized in the glycolysis. In addition, KB are taken up via MCT1 and MCT2 into the neurons, where they directly activate the TCA cycle and suppress VGLUT activity by occupying the chloride-binding site^37^, leading to vesicles with less neurotransmitter and reduction of glutamate release (Fig. 8B_1_). Inhibition of MCT1 and MCT2 under ketogenic conditions leads to an increase in the epileptiform activity, which is primarily mediated by the blockage of KB uptake into neurons. In addition to this, we presume that a conversion of the remaining lactate to pyruvate in neurons might also contribute to the increase of epileptiform activity (Fig. 8B_2_).

Taken together, our results indicate that inhibition of lactate uptake via MCT1/MCT2 into neurons leads to a substantial attenuation in seizures in the cortex from mice fed on CD, when lactate is the major fuel for neurons. This suggests that MCT1 and/or MCT2 in cortical neurons and astrocytes may be potential therapeutic targets for treatment of epilepsy. However, under ketogenic conditions, when glucose and KB are the primary energy sources of neurons, inhibition of MCT1 and MCT2 leads to an increase in the epileptiform activity. This indicates that MCTs play different roles in neuroprotection under ketosis and under conventional diet.

## Methods

### Animals, ketogenic diet and brain slices

Procedures involving mice were approved by the Landesuntersuchungsamt Rheinland-Pfalz, Koblenz, Germany (23 177–07-G 13–2–086). All experiments were performed in accordance with the relevant guidelines and regulations. The animals were maintained on a 12 h day/night cycle at constant room temperature with *ad libitum* access to water and standard mouse fodder in the animal facility of the University of Kaiserslautern. On month old mice (C57BL/6) were split randomly into two groups to follow control diet (CD; Cat N. 1314TPF, Altromin, Lage, Germany) or ketogenic diet (KD; Cat N. C1084, Altromin) for at least four weeks. The mice showed no obvious behavioural changes during daily handling. Blood glucose and BHB concentrations were measured directly after sacrificing the animal, using a precision Neo meter (Abbott Diabetes Care Ltd., Wiesbaden, Germany). Acute cortical slices were prepared from mice, 60–85 days old, which were killed by cervical dislocation and then decapitated. Brains were quickly transferred to an ice cold preparation solution containing (in mM): NaCl 25.2, KCl 2.5, CaCl_2_ 1.2, MgCl_2_ 5.0, Glucose 10, NaHCO_3_ 26, NaH_2_PO_4_ 1.2 and sucrose 176, bubbled with 5% CO_2_/95% O_2_, pH at 7.4. Cortical slices were obtained at a thickness of 150 µm using a vibratome (VT 1000, Leica, Darmstadt, Germany). Slices were incubated in artificial cerebrospinal fluid (aCSF) consisting of (in mM): NaCl 125, KCl 2.5, CaCl_2_ 0.5, MgCl_2_ 2.5, Glucose 25, NaHCO_3_ 26, and NaH_2_PO_4_ 1.25, gassed with 5% CO_2_/95% O_2_, at 30 °C for 1 hour to recover. After recovery, slices were stored in aCSF at RT up to 9 h until used for experiments. To perform all experiments with respect to circadian rhythm the mice and the food were weighted three times a week at the same time each day (11 am). The sacrifice of the animals, blood testing and brain extraction were always performed between 8 and 10 am.

### Preparation of mouse primary astrocyte cultures

Primary cultures of astrocytes were prepared from whole cerebral cortex of neonatal C57BL/6 mice (P0–P2). In brief, the mice were decapitated and the cerebrum was moved to a Petri dish containing ice-cold preparation solution containing (in mM): 120 NaCl, 5.4 KCl, 0.8 MgCl_2_, 25 Tris-HCl and 15 D-glucose. The meninges, olfactory bulb, cerebellum and hippocampus were carefully removed and then the cortex tissue was transferred into fresh cold preparation solution, then this solution was aspirated and the tissue incubated with 0.5% trypsin previously warmed up for 5 min. The trypsination was stopped by adding culture medium DMEM. In this medium, cells were singularized by pipetting up and down several times, followed by a centrifugation at 800 rpm for 7 min. After centrifugation, the medium was removed and the cells treated with 0.005% of DNAse and centrifuged once more at the same conditions. The supernatant was discarded and cells were resuspended in DMEM medium supplemented with penicillin 100 units/ml, streptomycin 0.1 mg/ml, 17 mM NaHCO_3_ and 10% horse serum. Cells were seeded on PDL-coated glass coverslips and incubated in a CO_2_ cell culture incubator for 1 h to allow proper cell attachment to the surface of the glass. After that 2 mL of DMEM supplemented medium was added to every petri dish. Cultured cells were kept with 5% CO_2_ and 95% atmospheric air at 37 °C. Medium was changed every two days until use. Cells were used for experiments after 10–20 days in culture.

### Fluorescence imaging

All imaging experiments were performed with a confocal laser-scanning microscope (LSM 700, Carl Zeiss, Oberkochen, Germany) at RT. Ca^2+^-imaging was performed using a 488 nm solid-state laser, short pass emission filter (SP 640 nm), a variable secondary dichroic mirror (VSD 595 nm) and a scanning frequency of 0.33 Hz. H^+^-imaging was performed using a 555 nm laser beam, SP 640 nm, VSD 590 nm and a scanning frequency of 0.2 Hz. Cortical slices were loaded with 5 µM Fluo-4-AM and 0.02% pluronic acid for Ca^2+^-imaging, and with 14 µM SNARF-5-AM for H^+^-imagining, in aCSF for 1 hour at 30 °C with constant CO_2_ recirculation and protected from the light. In order to keep the experiments comparable the laser intensity was kept constant for all experiments. All experiments were performed in the cortical region, comprising the primary motor area (layers 2/3 and 5) and the primary somatosensory area (layers 2/3, 4 and 5).

Changes in intracellular Ca^2+^ levels were recorded in CO_2_/HCO_3_^−^-containing solution as standard buffer (in mM): NaCl 125, KCl 2.5, CaCl_2_ 2.0, MgCl_2_ 1.0, Glucose 25, NaHCO_3_ 26, NaH_2_PO_4_ 1.25, and HEPES 10, constantly bubbled with 5% CO_2_/95% O_2_, pH at 7.4. To induce epileptiform activity, the samples were exposed to Mg^2+^-free standard buffer, supplemented with 20 µM bicuculline (0 Mg^2+^/BIC). At the end of each experiment, a pulse of 500 µM ATP or 250 µM L-Glutamate was applied to confirm that the cells could still show Ca^2+^ responses and are viable. All cells considered for analysis responded to both stimuli (0 Mg^2+^/BIC and ATP or L-Glutamate). Ca^2+^ variations recorded were expressed as the relative fluorescence intensity (R.F.I (%) = (F/F_0_)*100). F is the intensity of the fluorescence emission recorded along the experiment, and F_0_ is the fluorescence intensity during the first 60 sec of the experiment. The noise was calculated over the first 60 sec of each measurement and the threshold was defined as two times the noise. Astrocytes were considered to respond to stimulations when the amplitude of the exhibited transients was higher than the threshold (indicated with * in the results). The frequency was calculated as the number of peaks during the time of stimulation (# of peaks/min). The increase in the difference of the Ca^2+^ transient frequency was determined by the subtraction of the frequency evoked spontaneously during application of standard buffer to the frequency induced by application of 0 Mg^2+^/BIC.

H^+^-imaging recordings were performed in CO_2_/HCO_3_^−^-containing standard buffer. For preparation solutions containing KB or lactate, NaCl was replaced by an equivalent amount of sodium-L-lactate, DL-β-hydroxybutyric acid sodium salt and lithium acetoacetate, respectively. At the end of each measurement a pulse of CO_2_/HCO_3_^−^-free standard buffer was applied. In this solution NaHCO_3_ was replaced by an equivalent amount of NaCl and aeration of CO_2_/O_2_ was stopped. The ratio of SNARF emissions (F < 590 nm/F > 590 nm) was calibrated using nigericin/30 mM K^+^ and converted into proton concentration (Fig. S5). Cells were confirmed to be astrocytes by staining with sulforhodamine (SR101), as previously described^41, 42^. After Ca^2+^- and H^+^-measurements, slices were loaded with 0.5 µM SR 101 in standard buffer at RT for 15 min, followed by a washing step with standard buffer in the absence of SR101 for 15 min. SR101 was excited at 555 nm, and emission collected at 603 nm (Fig. S6).

### Buffer capacity and proton fluxes

The intrinsic buffer capacity β_i_ was calculated from the maximal changes in pH_i_ (amplitude) recorded when changing from CO_2_/HCO_3_^−^-containing standard buffer to CO_2_/HCO_3_^−^-free standard buffer with ${{\rm{\beta }}}_{{\rm{i}}}={\rm{\Delta }}{[{{{\rm{HCO}}}_{3}}^{-}]}_{{\rm{i}}}{/{\rm{\Delta }}\mathrm{pH}}_{{\rm{i}}}$. The CO_2_/HCO_3_^−^-dependent buffer capacity ${{\rm{\beta }}}_{{{\rm{CO}}}_{2}}$ was calculated from ${{\rm{\beta }}}_{{{\rm{CO}}}_{2}}=2.3\ast [{{{\rm{HCO}}}_{3}}^{-}]$. The bicarbonate concentration was calculated from the pH_i_ measured and based on the Henderson-Hasselbach equation, assuming a [CO_2_] of 0.00835 mM and 1.3 mM, in CO_2_/HCO_3_^−^-free and CO_2_/HCO_3_^−^-containing standard buffer, respectively. The total buffer capacity β_T_, was calculated as the sum of β_i_ and ${{\rm{\beta }}}_{{{\rm{CO}}}_{2}}$. The net H^+^ flux J_H_ (mM/min), defined as the net transport of acid and/or base equivalents across a cell membrane, was calculated as the product of the rate of pH_i_ change during application of substrate and the intrinsic buffer capacity β_T_^43^.

### Protein extraction and immunoblot analysis

Brain cortex lysate was produced by sonication in 400 µL of 2% SDS containing protease inhibitors (Complete Mini EDTA-free, Roche Diagnostics GmbH, Mannheim, Germany). The lysates were centrifuged at 4 °C, 1400 × g for 5 min and the supernatants were collected. Protein content was determined by IR spectrometry (Direct Detect Spectrometer, Merck Millipore, Darmstadt, Germany). 50 µg of protein lysate were loaded onto a 4–20% Run Blue SDS gel and electrotransferred onto nitrocellulose membranes. Blots were probed with anti-MCT1 rabbit polyclonal antibody (1:200; Merck Millipore), anti-MCT2 mouse monoclonal antibody (1:200, Santa Cruz, Heidelberg, Germany), anti-MCT4 rabbit polyclonal antibody (1:200; Merck Millipore), anti-GLUT1 rabbit polyclonal antibody (1:500, Merck Millipore) or anti-GLUT3 rabbit monoclonal antibody (1:1000, Abcam, Cambridge, UK) and peroxidase conjugated goat anti-rabbit IgG secondary antibody (1:2000 for MCT1, MCT4, GLUT3; 1:4000 for GLUT1; Santa Cruz) or peroxidase conjugated goat anti-mouse IgG secondary antibody (1:4000; Santa Cruz).

### Mass spectrometry

Metabolite extraction was carried out based on protocols published previously^44, 45^. In brief, slices were incubated in solution containing 2 mM of BHB or BHB-free solution for three hours before being placed in liquid nitrogen. 14 slices (equivalent to 42 mg of tissue) were pulverized with a mortar and pestle and immediately collected in tubes at −80 °C. 1 mL of extraction solvent, containing 40% acetonitrile, 40% methanol and 20% distilled water, was added to each sample, mixed, spun down at 4 °C and 14000 rpm for 10 min, and the supernatant transferred to a fresh tube. The extraction solvent was evaporated to dryness using a speed vacuum overnight at −80 °C. The dry extracts were reconstituted in 50 µL mobile phase (0.1% formic acid + 10% methanol in distilled water), pH adjusted to < 7.0, centrifuged, and the supernatant was taken. The analysis of the samples was performed using an Agilent HPLC system (Modular system series 1100, Fa. Agilent Technologies, Waldbronn, Germany) coupled with a tandem mass spectrometer (MS; QT4000, Fa. AB Sciex GmbH, Darmstadt, Germany). The analysis was performed with three different batches of mice (3 CD and 3 KD animals). Calibration curves were produced using standards at 10, 100, 200, 500, 1000 and 2000 µM for BHB and 100, 200 and 500 µM for lactate. The area under the curve for each standard application was calculated and plotted against the amount of substrate tested. A linear and a quadratic fit were adjusted for BHB and lactate, respectively (Fig. S7). The total amount of metabolites in cortical slices was given in µg/mg of tissue fresh weight (f.w.). The conversion from µM to µg/mg of tissue f.w. was carried out using the equation $T\,({\rm{\mu }}g/\mathrm{mg})=\frac{{\rm{X}}\ast {{\rm{MW}}}_{{\rm{Substrate}}}\ast {{\rm{V}}}_{{\rm{e}}}}{{{\rm{W}}}_{{\rm{slice}}}}\ast {10}^{-6}$. X is the amount of BHB or lactate (µM) expressed in M units. MW_Substrate_ is the molecular weight of the anions in g/mol (104.11 g/mol for BHB, 89.07 g/mol for lactate). V_e_ is the elution volume used to dissolve each sample in litres (L). W_slice_ is the weight corresponding to one slice (mg), which is 3 mg.

### Chemicals

The inhibitor AR-C155858, tetrodotoxin citrate (TTX) and sulforhodamine 101 (SR101) were acquired from Tocris, Wiesbaden-Germany, Abcam Biochemicals, Cambridge, UK, and Baseclick, Tutzing, Germany, respectively. Fluo-4-AM, SNARF-5-AM, and pluronic acid were obtained from Invitrogen. The fluorescent dyes, pluronic acid, AR-C155858 and SR101 were dissolved in DMSO.

### Data analysis and statistics

To analyse imaging experiments, regions of interest (ROIs) were manually drawn around the cellular body, using ImageJ. For Ca^2+^-imaging, the fluorescent signal was exported as ASCII file, and further analysed using OriginPro 8.6 (OriginLab Corporation, USA). For H^+^-imaging, the ImageJ ratio plus plugin was employed to calculate the fluorescence intensity (F.I.) ratio and exported as ASCII. Matlab (MatWorks Inc., USA) was used to convert the F.I. ratio to pH values and [H^+^], and analysed with OriginPro 8.6. Statistical values are presented as means ± standard error of the average (S.E.M). For calculation of significance in differences, student’s t-test or, if possible, a paired t-test was used. Gaussian distribution of the data was assumed, since the number of cells considered in each experiment was always higher than 30, which is big enough to assume Gaussian distribution^46^. In the particular cases where the number of cells is unknown (like it was the case for western blot and mass spectrometry experiments) samples from at least three different animals were measured for each condition. In that case a Jarque-Bera test was performed to confirm normality of the data, which again allows assumption of normal distribution^46^. In the figures shown, a significance level of p ≤ 0.05 is marked with *p ≤ 0.01 with ** and p ≤ 0.001 with ***.

## Electronic supplementary material

Supplementary Figures
