# Supplementary material for: Reduction of epileptiform activity in ketogenic mice: The role of monocarboxylate transporters
Source: Sci Rep. 2017 Jul 7;7:4900. doi: 10.1038/s41598-017-05054-0 (PMC5501801; doi:10.1038/s41598-017-05054-0)
Supplement: Supplementary file 1 — Supplementary Figures [file 41598_2017_5054_MOESM1_ESM.pdf]

Reduction of epileptiform activity in ketogenic mice:
The role of monocarboxylate transporters

Linda S. Forero-Quintero, Joachim W. Deitmer and Holger M. Becker

Division of General Zoology, Department of Biology, University of Kaiserslautern, P.O. Box 3049, D-67653 Kaiserslautern, Germany

SUPPLEMENTARY FIGURES

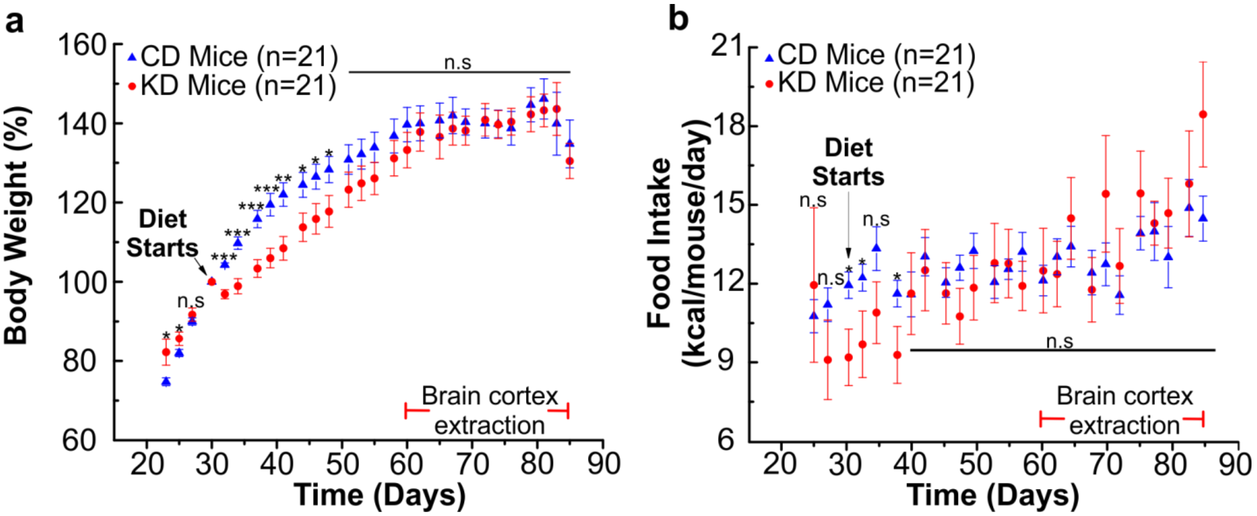

Figure S.1 | Effects of control (CD) and ketogenic (KD) diets on wild-type mice. Effects of control (CD, blue triangles) and ketogenic (KD, red circles) diets on **(a)** body weight and **(b)** food intake in wild-type mice. Statistical values are presented as means ± S.E.M and significance was tested with a paired Student's t-test. Number of mice (n) used are indicated in the plots.

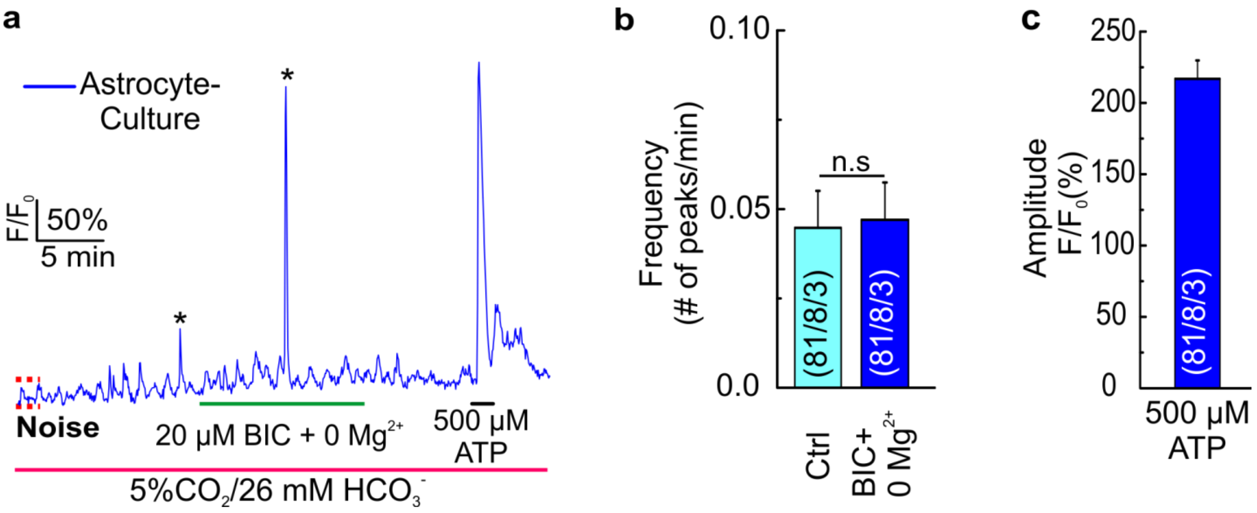

Figure S.2 | Epileptiform activity induced in pure astrocyte culture. (a) Changes in astrocytic Ca^{2+} signals induced by epileptiform activity (0 Mg^{2+}/BIC) in cultured cortical astrocytes in 5 % CO_2 / 26 mM HCO_3^--buffer. One short pulse of 500 µM ATP was given at the end to verify astrocytic calcium response. **(b)** Frequency of the Ca^{2+} transients occurred spontaneously (cyan bar) and induced by 0 Mg^{2+}/BIC (blue bar) in CO_2 /HCO_3^--buffer. **(c)** Amplitude of the Ca^{2+} transients induced by ATP in cultured cortical astrocytes. Statistical values are presented as means ± S.E.M and significance was tested with a paired Student's t-test. Number of astrocytes/culture/mice used are indicated in the bar plots.

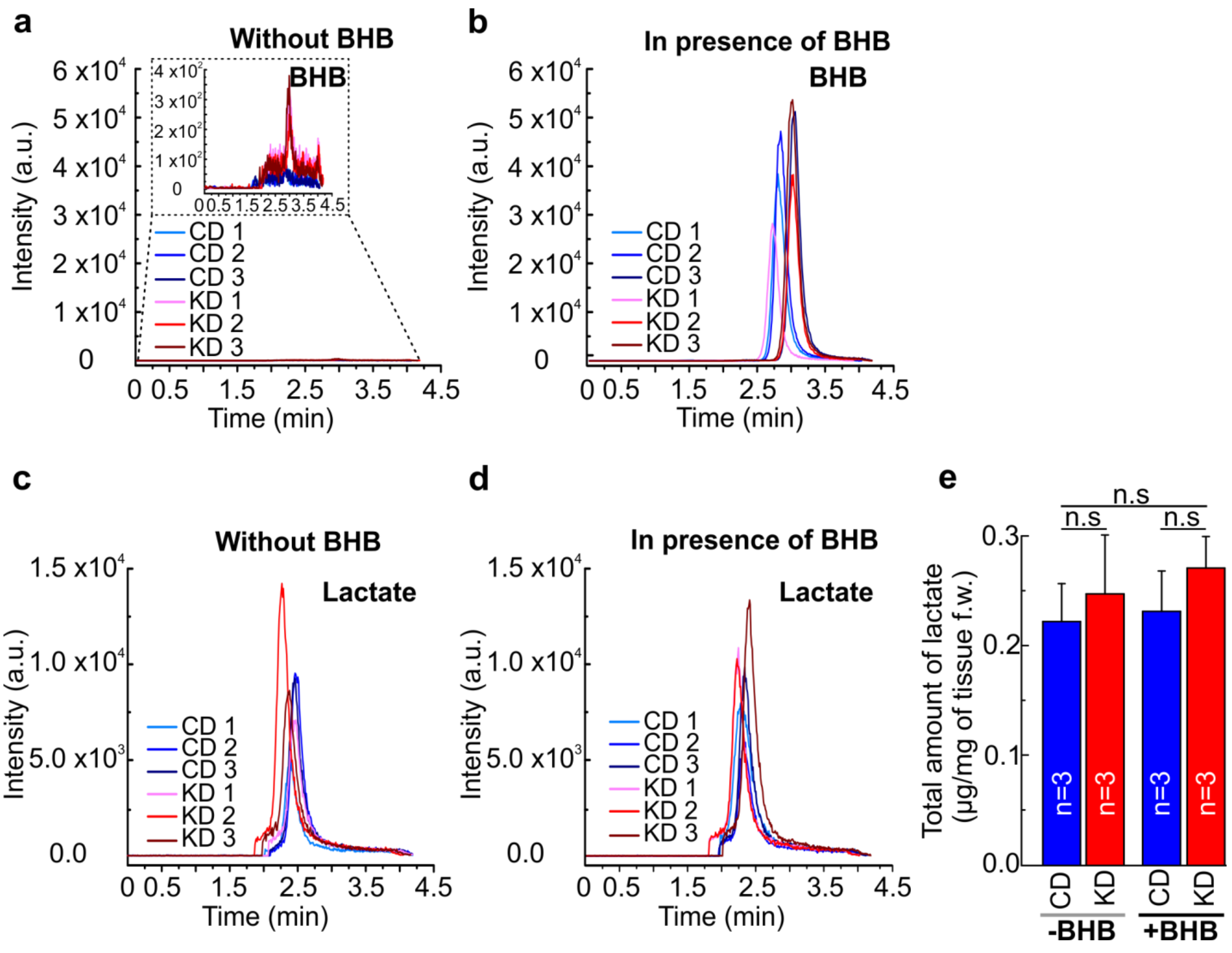

Figure S.3 | Quantification of BHB and lactate in cortical slices measured by mass spectrometry. Extracted ion chromatograms of BHB **(a)** in the absence and **(b)** in the presence of BHB during the sample preparation. The inset in **(a)** presents the same data but with amplified scale in *y* axis to show differences. BHB was extracted from cortical slices of mice that followed CD (blue scale traces) or KD (red scale traces). For quantification of the total amount of BHB in cortical slices see Fig. 2G. Extracted ion chromatograms of lactate **(c)** in the absence and **(d)** in the presence of BHB during the sample preparation. Lactate was extracted from cortical slices of mice that followed CD (blue scale traces) or KD (red scale traces). **(e)** Quantification of the total amount of lactate in slices from CD (blue bars) and KD (red bars) mice, in the absence and in the presence of BHB. Statistical values are presented as means ± S.E.M and significance was tested with a paired Student's t-test. Number of mice (n) used are indicated in the bar plots.

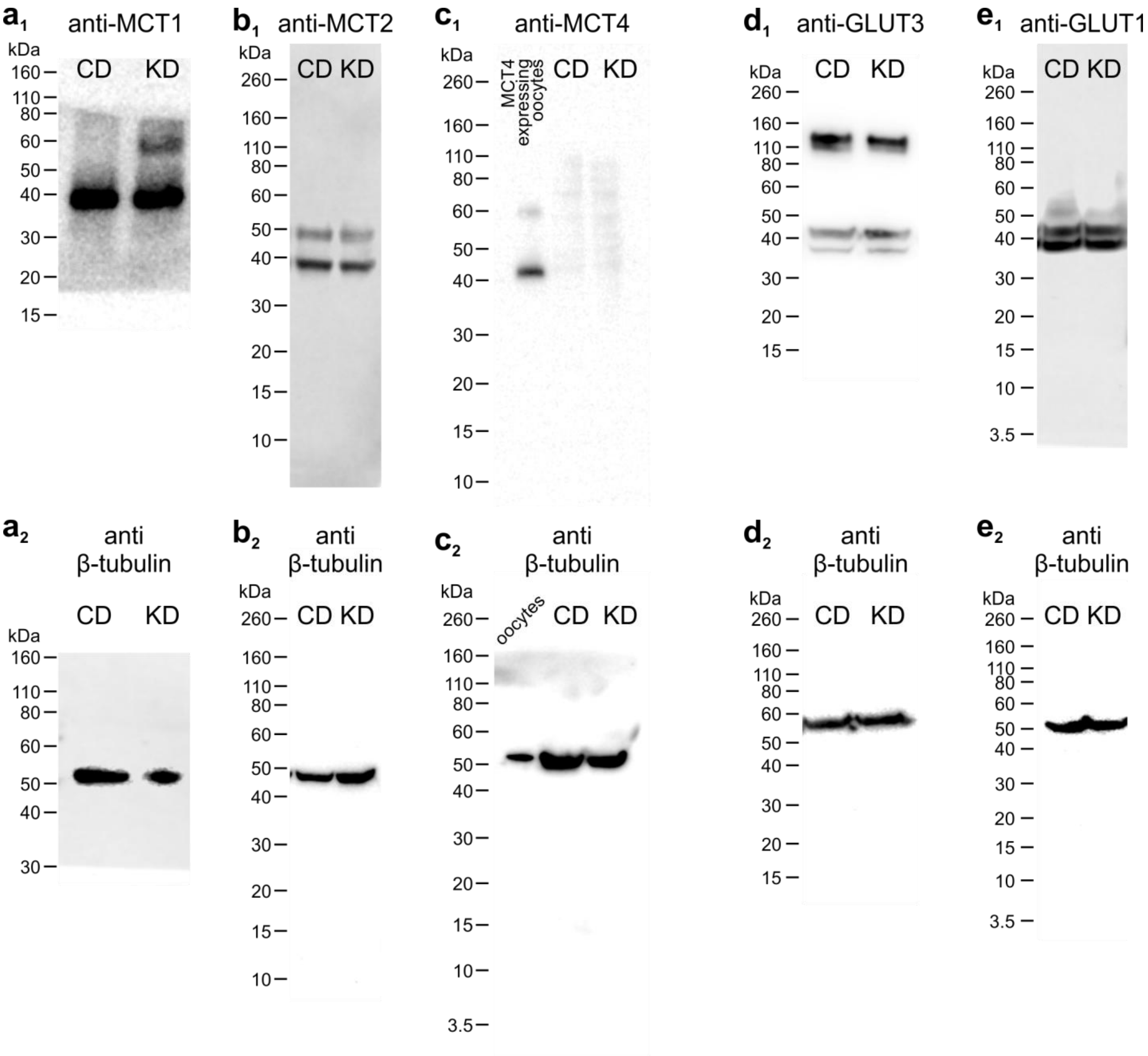

Figure S.4 | Full-length western blots for MCT1 ($\mathbf{a_1}$), MCT2 ($\mathbf{b_1}$), MCT4 ($\mathbf{c_1}$), GLUT3 ($\mathbf{d_1}$) and GLUT1 ($\mathbf{e_2}$) from cortex lysates from mice fed with control (CD) and ketogenic diet (KD), respectively. For MCT4 a lysate of MCT4-expressing *Xenopus* oocytes was used as positive control (c_1, c_2). After analysis the blots were labelled with β-tubulin as loading control ($\mathbf{a_2}$, $\mathbf{b_2}$, $\mathbf{c_2}$, $\mathbf{d_2}$, $\mathbf{e_2}$).

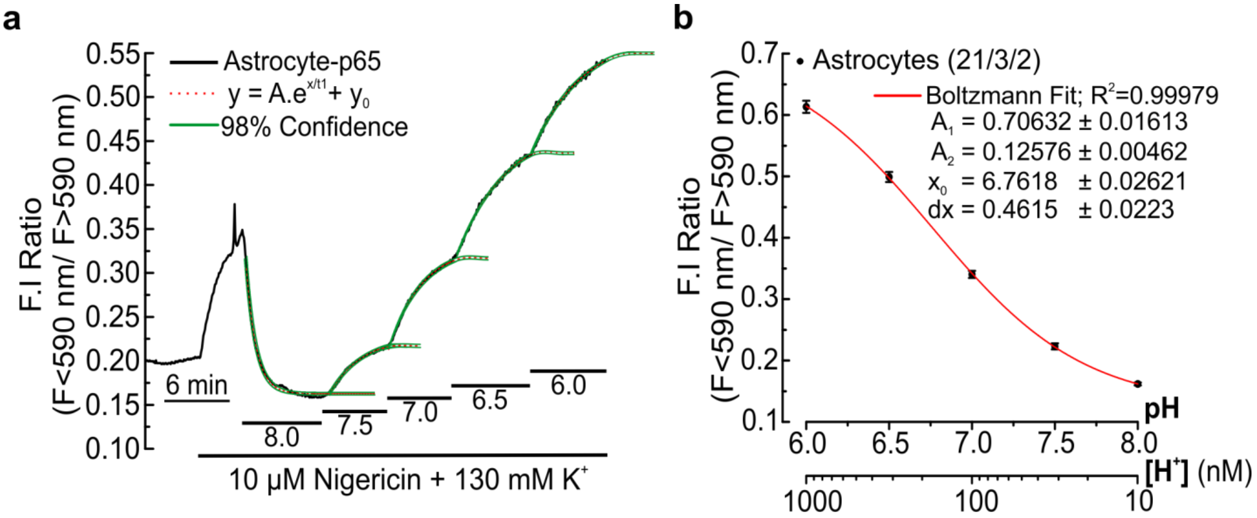

Figure S.5 | Calibration of SNARF-5 in cortical astrocytes in tissue slices. (a) Calibration of the fluorescence intensity ratio (F.I. Ratio) signal in the presence of nigericin and 130 mM K^+, at pH 8.0, 7.5, 7.0, 6.5 and 6.0. An exponential equation was used to calculate the maximum steady state for each pH application, as indicated in the plot (red trace), the confidence was 98 % (gray trace). **(b)** F.I. Ratio against pH values and its respective H^+ concentration scale. The number of astrocytes/slices/animals used in the experiments are indicated in the plot. A Boltzmann fit (black) was adjusted to calculate the parameters of conversion (shown in the plot). The coefficient of correlation (R^2) was 0.99979. pH and H^+ concentration were calculated as, $\mathrm{pH} = \mathrm{x}_0 + \mathrm{dx} * \ln \frac{\mathrm{A}_1 - \mathrm{A}_2}{\mathrm{R} - \mathrm{A}_2} - 1$ and $H^+\ (nM) = 10^{-pH} * 10^9$. Statistical values are presented as means ± S.E.M.

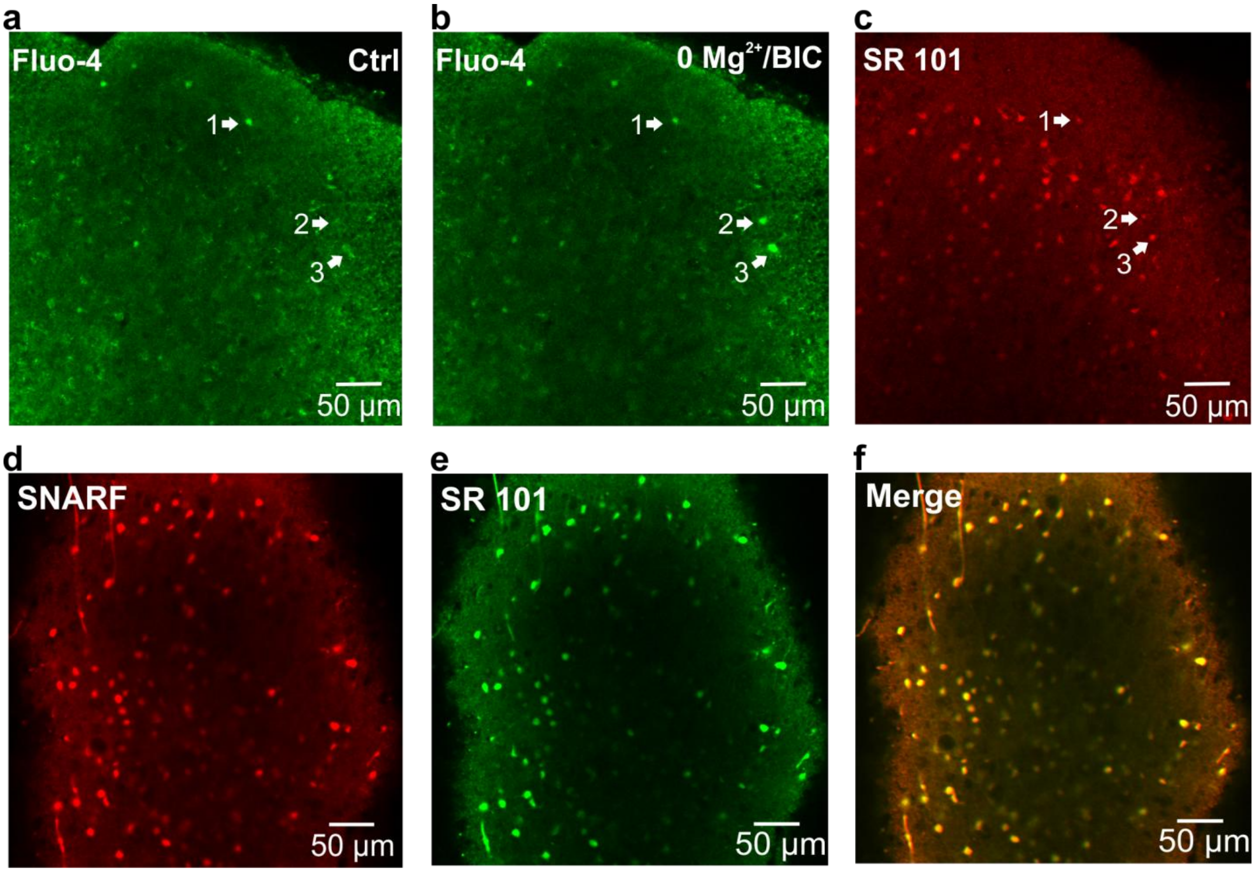

Figure S.6 | Correlation between astrocytes labeled with the Ca^{2+} and H^{+} sensitive dyes and the astrocyte-specific antibody SR 101 in cortical slices. Image of an area of Fluo-4 loaded (Ca^{2+}-sensitive dye) acute cortical tissue slice **(a)** at the beginning of the experiment and **(b)** at some time point during bath application of 0 Mg^{2+}/BIC in the standard saline for slices. The white arrows point some cells responding to the stimulus at the specific time point. **(c)** Specific astrocytic identification with sulforhodamine (SR 101), fluorescence taken from the same area. The white numbered arrows point some of the astrocytes that responded to 0 Mg^{2+}/BIC and were labeled positively with SR 101. **(d)** Image of an area of SNARF-loaded acute cortical tissue slice. **(e)** Specific astrocytic identification with SR 101, fluorescence taken from the same area. **(f)** An overlay of SNARF-loaded and SR101-positive astrocytes appears in orange-yellow. The pictures were taken with a confocal microscope LSM 700, with the following characteristics: objective= W Plan-Apochromat 20X/1.0 DIC M27 75 mm, and zoom=0.7.

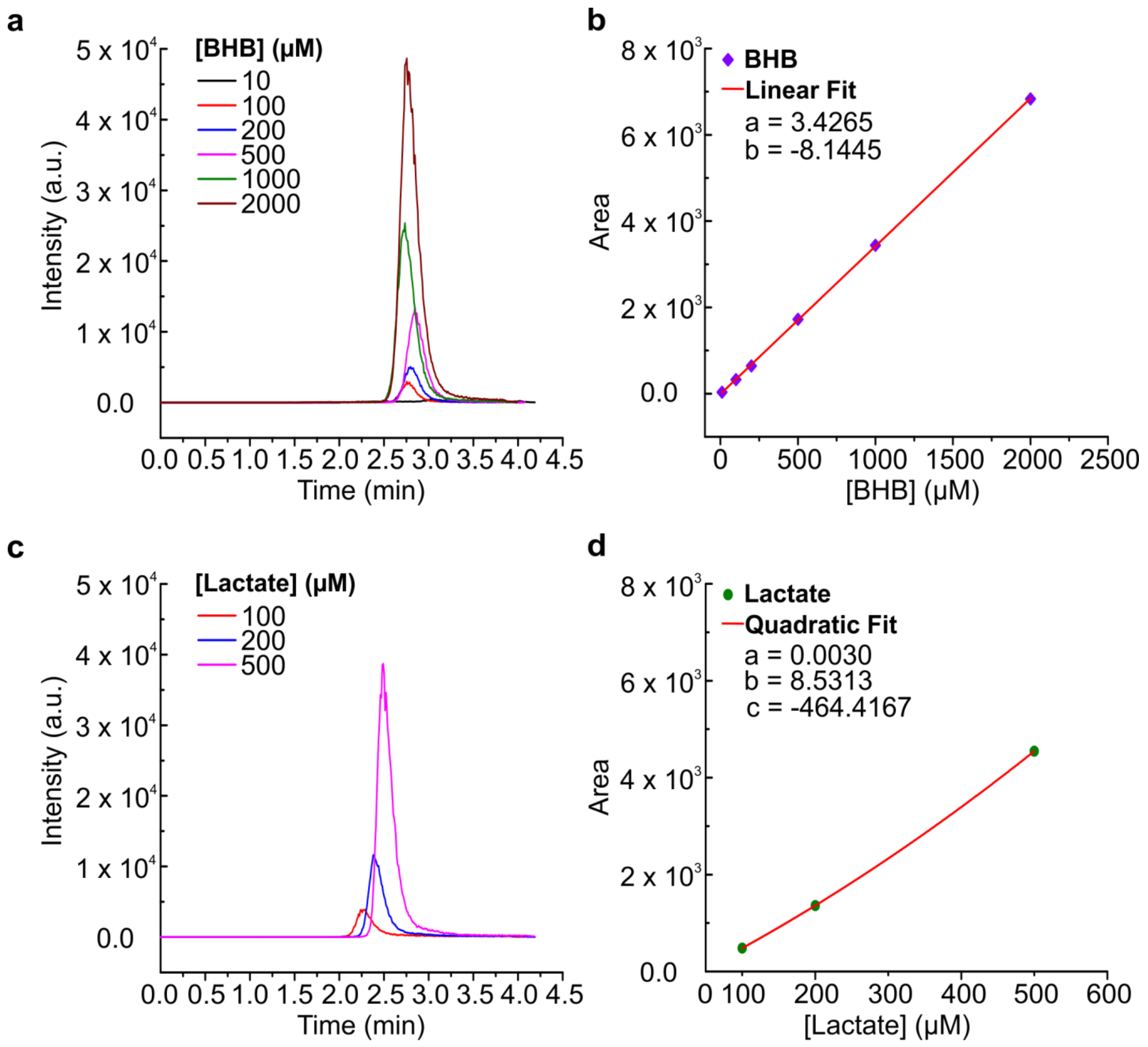

Figure S.7 | Mass spectrometry calibrations for BHB and lactate. Intensity of the peaks against retention time for different standard concentrations of **(a)** BHB and **(c)** lactate (10 (black), 100 (red), 200 (blue), 500 (pink), 1000 (green) and 2000 (red wine) µM). Calibration curves for **(b)** BHB and **(d)** lactate, plotted as the area under the curve vs. substrate concentration. The calibration fit for each substrate is shown in red (a linear fit for BHB, and a quadratic fit for lactate). The coefficient of correlation (R^2) were 0.99997 and 1.0 for BHB and lactate, respectively. The parameters obtained from the calibration are shown in the plot.
